# Supplementary material for: Exploring Genome-Wide Mutation Dynamics and Bacterial Cellulose Impairment in Komagataeibacter intermedius Cultivated Under Agitation Stress
Source: Comput Struct Biotechnol J. 2026 Mar 18;35(1):0009. doi: 10.34133/csbj.0009 (PMC13068021; doi:10.34133/csbj.0009)
Supplement: Supplementary 1 — Additional File 1 Figs. S1 to S12 Tables S1 and S2 Additional Files 2 to 5 [file csbj.0009.f1.zip › Additional File 3.zip!/READ ME_Additional_File_3.docx]

READ ME FILE _ ADDITIONAL FILE 3

Dear Reader,

Once un-zipped you will see two folders “**Parental strain**” and “**Additional File 3**”. The folder named “**Parental strain**” is to be considered part of the Additional File 3 materials, but it has been separated to have an easier access to the data.

The “**Additional File 3**” folder contains the Breseq raw outputs related to the agitated cultivation rounds.

All the folders present in “**Additional File 3**”, are relative to a specific lineage and round: for example, A2 🡪 A = lineage, 2= round 2. Inside one of this folders (e.g.: A2) there would be three different type of files ( the same applies for the Parental Strain folder).

1) **evidence.zip**. This zipped file contains the reads alignment of the mutations detected by Breseq in the sequenced genome.

2) I**ndex.html.** This is the main output of Breseq. The html files report all the predicted mutations and junction events.

3)**output.gd** is the output, in gd format, regarding the mutations presented in the file index.html. The output.gd file does not report information on the annotation of the regions involved in a mutation.

Useful instructions and tips on how to interpretate the raw data are freely available on the GitHub page of Breseq. (<https://github.com/barricklab/breseq>)
